# Kidney ACE2 expression: Implications for chronic kidney disease

**DOI:** 10.1371/journal.pone.0241534

**Published:** 2020-10-30

**Authors:** Nicholas Maksimowski, Vanessa R. Williams, James W. Scholey

**Affiliations:** 1 Institute of Medical Science, University of Toronto, Toronto, Ontario, Canada; 2 Department of Physiology, University of Toronto, Toronto, Ontario, Canada; 3 Division of Nephrology, University Health Network, Toronto, Ontario, Canada; 4 Toronto General Hospital Research Institute, University Health Network, Toronto, Ontario, Canada; International University of Health and Welfare, School of Medicine, JAPAN

## Abstract

Angiotensin-converting enzyme 2 (ACE2) has been implicated in the pathogenesis of chronic kidney disease (CKD) and is a membrane receptor for severe acute respiratory syndrome coronavirus 2 (SARS-CoV-2), the virus responsible for coronavirus disease (COVID-19), whereas transmembrane protease, serine 2 (TMPRSS2) is involved in viral attachment. Together, tissue expression of ACE2 and TMPRSS2 may determine infection. Sex, age, body mass index (BMI), and CKD are clinical risk factors for COVID-19 severity, but the relationships between kidney ACE2 and TMPRSS2 expression and these clinical variables are unknown. Accordingly, we obtained renal tubulointerstitial and glomerular microarray expression data and clinical variables from healthy living donors (HLD) and patients with CKD from the European Renal cDNA Bank. ACE2 expression was similar in the tubulointerstitium of the two groups, but greater in females than males in HLD (*P* = 0.005) and CKD (*P* < 0.0001). ACE2 expression was lower in glomeruli of CKD patients compared to HLD (*P* = 0.0002) and lower in males than females. TMPRSS2 expression was similar in the tubulointerstitium but lower in glomeruli of CKD patients compared to HLD (*P* < 0.0001). There was a strong relationship between ACE2 and TMPRSS2 expression in the glomerulus (*r* = 0.51, *P* < 0.0001). In CKD, there was a relationship between tubulointerstitial ACE2 expression and estimated glomerular filtration rate (*r* = 0.36, *P* < 0.0001) and age (*r* = -0.17, *P* = 0.03), but no relationship with BMI. There were no relationships between TMPRSS2 expression and clinical variables. Genes involved in inflammation (*CCL2*, *IL6*, and *TNF*) and fibrosis (*COL1A1*, *TGFB1*, and *FN1*) were inversely correlated with ACE2 expression. In summary, kidney expression of ACE2 and TMPRSS2 differs in HLD and CKD. ACE2 is related to sex and eGFR. ACE2 is also associated with expression of genes implicated in inflammation and fibrosis.

## Introduction

Angiotensin-converting enzyme 2 (ACE2) modifies angiotensin peptide metabolism and affects the progression of chronic kidney disease (CKD) [1]. In the kidney, ACE2 is predominantly expressed in proximal tubules and glomerular podocytes [2, 3]. ACE2 converts angiotensin (Ang) I to Ang-(1–9) and Ang II to Ang-(1–7) [4, 5]. As a proinflammatory and profibrogenic peptide, Ang II is a major contributor to the progression of CKD [6, 7]. Conversely, Ang-(1–7) acts as an antagonist to the harmful actions of Ang II [8–10]. Thus, ACE2, which is capable of reducing Ang II and promoting Ang-(1–7), serves as a key protective enzyme by reducing oxidative stress, inflammation, and fibrosis [11].

ACE2 has gained considerable attention for its role as the functional host cell-surface receptor for the virus, severe acute respiratory syndrome coronavirus 2 (SARS-CoV-2), responsible for coronavirus disease (COVID-19) [12]. Along with transmembrane protease, serine 2 (TMPRSS2), which cleaves (i.e., primes) the SARS-CoV-2 spike protein and facilitates membrane fusion and viral entry, ACE2 is a critical component of SARS-CoV-2 cell entry mechanisms [12–14]. Multiple risk factors for COVID-19 have emerged. Of particular interest is that CKD has been identified as a risk factor for COVID-19. Patients with CKD have more severe outcomes, and acute kidney injury is commonly observed in patients with COVID-19 [15–18]. Autopsies of patients with COVID-19 have revealed evidence of kidney tropism and direct renal infection [19, 20]. Other risk factors, such as male sex, older age, and higher body mass index (BMI), are associated with poorer prognosis, increased hospitalizations, and higher mortality in people with COVID-19 [21–25].

Relatively few studies have characterized ACE2 in human kidneys despite the significance of ACE2 in CKD pathogenesis. In the tubular compartment, subjects with diabetic nephropathy (DN), IgA nephropathy (IgAN), membranous glomerulonephropathy (MGN), or hypertensive nephropathy (HN) had lower ACE2 expression than healthy controls [26–29]. Glomerular ACE2 expression was lower in patients with DN or IgAN compared to healthy controls [26–28], whereas glomerular ACE2 expression in patients with other forms of CKD—specifically, focal segmental glomerulosclerosis (FSGS), chronic allograft nephropathy, minimal change disease (MCD), MGN, and HN—was similar to that of control subjects [26, 28, 29]. The relationships between kidney ACE2 expression and clinical variables such as age, sex, and BMI have not been studied in healthy subjects or in CKD. Moreover, the relationship between kidney ACE2 expression and eGFR in CKD is not fully understood [28, 29].

Given the importance of ACE2 and TMPRSS2 in regard to SARS-CoV-2 infection and the relationship between CKD and COVID-19 severity, we compared tubulointerstitial and glomerular expression of ACE2 and TMPRSS2 in healthy living donors (HLD) and subjects with CKD from the publicly available European Renal cDNA Bank (ERCB; nephroseq.org, University of Michigan, Ann Arbor, MI). We then related ACE2 and TMPRSS2 mRNA expression in CKD to clinical variables. Finally, we explored relationships between the expression of ACE2 and the expression of genes implicated in inflammation and fibrosis.

## Materials and methods

### Data collection and study cohort

Clinical characteristics and pre-processed log2 median-centered microarray intensity values from the “Ju CKD Glom” [30] and “Ju CKD TubInt” [31] datasets were accessed from Nephroseq (v4; nephroseq.org, May 2020, University of Michigan, Ann Arbor, MI). Specifically, these datasets include microarray expression data generated from microdissected kidney glomerular and tubulointerstitial biopsy samples in the ERCB (S1 Dataset). The ERCB biopsies were obtained from patients after informed consent and with approval of the local ethics committees. Clinical and gene expression information from patients are accessible in a non-identifiable manner. From the open source dataset, subjects with DN, FSGS, HN, IgAN, lupus nephritis, MGN, MCD, thin basement membrane disease, tumor nephrectomy, and vasculitis were examined. Kidneys of HLD served as the control group. As per published reports, mRNA levels in the ERCB were derived using Affymetrix GeneChip Human Genome U133A 2.0 and U133 Plus 2.0 arrays [32].

### Statistics

Analyses were performed using GraphPad Prism 7 (GraphPad Software, San Diego, CA). Data are presented as the mean ± SEM, unless otherwise stated. Tubulointerstitial and glomerular median-centered log2 mRNA expression of ACE2 or TMPRSS2 from renal biopsy samples were compared in CKD and HLD, as well as in male and female subgroups. ACE2 or TMPRSS2 expression levels were correlated against estimated glomerular filtration rate (eGFR), age, and BMI. ACE2 expression was also correlated against expression of monocyte chemoattractant protein 1 (MCP-1 or *CCL2*), interleukin 6 (*IL6*), tumor necrosis factor alpha (*TNF*), collagen, type I, alpha 1 (*COL1A1*), transforming growth factor beta 1 (*TGFB1*), fibronectin (*FN1*), and TMPRSS2 in the CKD population. For comparisons between two groups, two-tailed *P* values were determined by χ^2^ tests for categorical variables and unpaired Student’s *t* test for continuous variables. For comparisons between more than two groups, *P* values were determined by one-way analysis of variance. Pearson’s correlation coefficient (*r*) with two-tailed *P* values were calculated. Linear regression was used to generate the line of best fit with 95% confidence intervals.

## Results

### Patient characteristics in HLD and CKD

There were 31 subjects in the HLD tubulointerstitial cohort (Table 1) including 10 females and 12 males. The sex was not specified for 9 subjects. The average age was 47.3 ± 2.4 years with a mean eGFR_MDRD_ of 104.3 ± 6.5 ml/min/1.73 m^2^. The CKD tubulointerstitial cohort (Table 1) included 170 subjects with a range of primary kidney diseases (Table 2) and included 75 females and 95 males. The average age was 46.8 ± 1.4 years with a mean eGFR_MDRD_ of 66.4 ± 2.9 ml/min/1.73 m^2^.

**Table 1:** Demographic and clinical characteristics of healthy living donors and patients with chronic kidney disease.

	Tubulointerstitium			Glomerulus		
	HLD	CKD	*P* value (CKD vs. HLD)	HLD	CKD	*P* value (CKD vs. HLD)
***n***	31	170		21	178	
**Sex (females/males)**	10/12	75/95	0.9054	9/12	85/93	0.8462
**Age (years)**	47.3 ± 2.4	46.8 ± 1.4	0.9008	47.2 ± 2.6	46.4 ± 1.3	0.8461
**BMI (kg/m**^**2**^**)**	23.7 ± 0.1	25.7 ± 0.4	0.5043	23.7 ± 0.1	25.7 ± 0.4	0.4993
**Mean BP (mm Hg)**	93.3 ± 10.7	100.2 ± 1.3	0.5177	93.3 ± 10.7	100.0 ± 1.2	0.5297
**SCR (mg/dl)**	0.82 ± 0.06	1.65 ± 0.11	0.0051	0.83 ± 0.06	1.58 ± 0.10	0.0077
**BUN (mg/dl)**	–	44. 5 ± 3.3	–	–	42.4 ± 2.8	–
**eGFR (ml/min/1.73 m**^**2**^**)**	104.3 ± 6.5	66.4 ± 2.9	< 0.0001	105.4 ± 6.7	67.6 ± 2.9	< 0.0001

BMI, body mass index; BP, blood pressure; BUN, blood urea nitrogen; CKD, chronic kidney disease; eGFR, estimated glomerular filtration rate; HLD, healthy living donor; SCR, serum creatinine. For continuous variables, values are presented as the mean ± SEM and *P* values were determined by Student’s *t* tests. For categorical variables, *P* values were determined by χ^2^ tests.

**Table 2:** Distribution of chronic kidney disease patient samples from each renal compartment across each disease type.

	Tubulointerstitium	Glomerulus
**DN**	17	12
**FSGS**	17	25
**HN**	20	15
**IgAN**	25	27
**LN**	32	32
**MCD**	14	14
**MGN**	18	21
**TBMD**	6	3
**TN**	–	6
**Vasculitis**	21	23

DN, diabetic nephropathy; FSGS, focal segmental glomerulosclerosis; HN, hypertensive nephropathy; IgAN, IgA nephropathy; LN, lupus nephritis; MCD, minimal change disease; MGN, membranous glomerulonephropathy; TBMD, thin basement membrane disease; TN, tumor nephrectomy.

There were 21 subjects in the HLD glomerular cohort (Table 1) including 9 females and 12 males, and the average age was 47.2 ± 2.6 years with a mean eGFR_MDRD_ of 105.4 ± 6.7 ml/min/1.73 m^2^. The CKD glomerular cohort (Table 1) included 178 subjects with a range of primary kidney diseases (Table 2). There were 85 females and 93 males, and the average age was 46.4 ± 1.3 years with a mean eGFR_MDRD_ of 67.6 ± 2.9 ml/min/1.73 m^2^.

### Comparison of ACE2 mRNA expression in HLD and CKD

**Fig 1:**
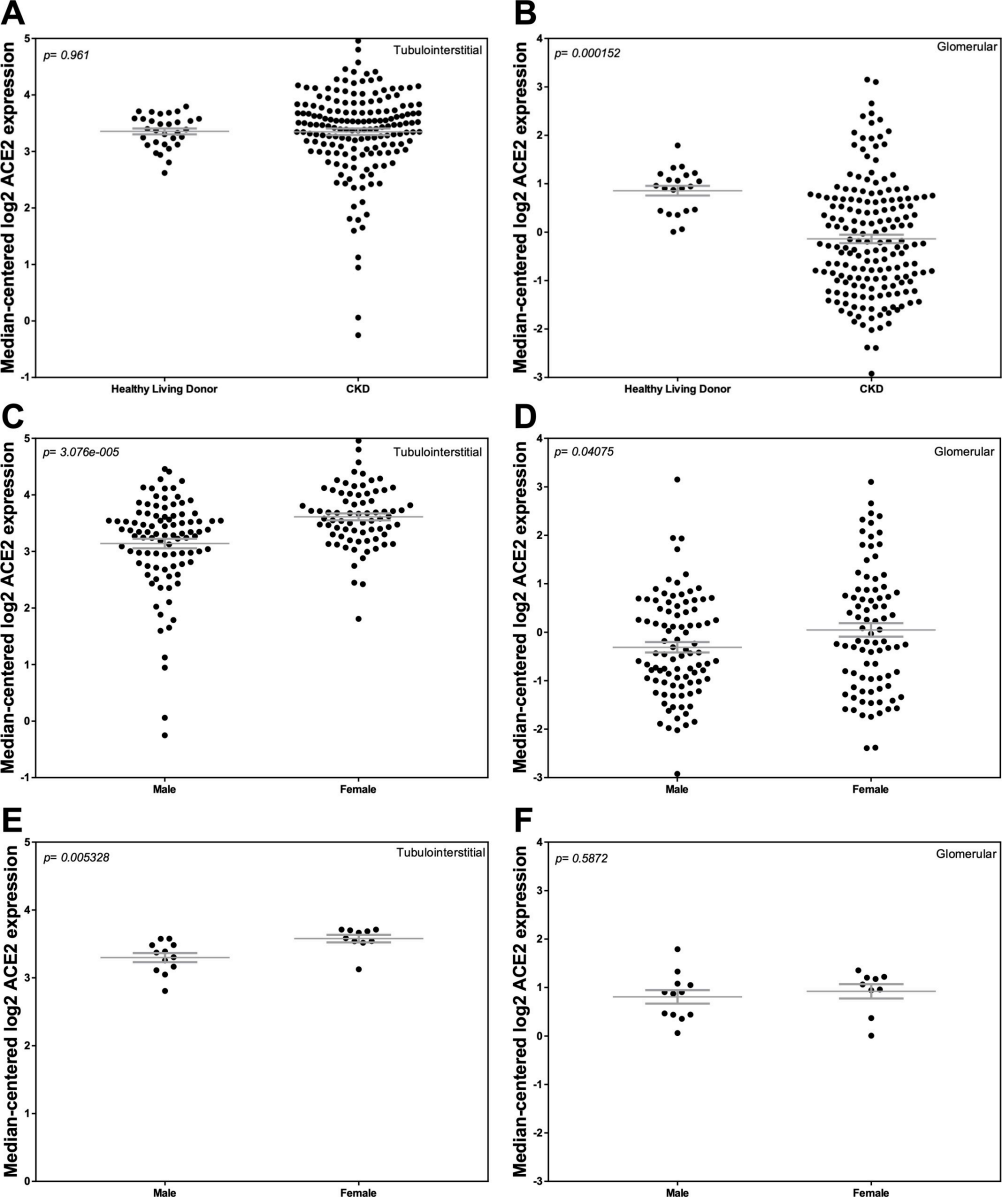
Tubular and glomerular Angiotensin-Converting Enzyme 2 (ACE2) mRNA expression in Chronic Kidney Disease (CKD) and Healthy Living Donors (HLD). (A, C, E) Tubular ACE2 mRNA expression in (A) HLD and subjects with CKD, (C) males and females with CKD, and (E) male and female HLD. (B, D, F) Glomerular ACE2 expression in (B) HLD and subjects with CKD, (D) males and females with CKD, and (F) male and female HLD. Values are the mean ± SEM (grey lines). *P* values were determined by Student’s *t* tests.

We compared ACE2 mRNA expression in both kidney compartments in the two cohorts and examined ACE2 mRNA expression based on sex. Tubulointerstitial ACE2 mRNA expression was similar between the two groups, with more variability in CKD (Fig 1A). ACE2 mRNA expression in the tubulointerstitium was greater in females compared to males in CKD (*P* < 0.0001; Fig 1C) and in HLD (*P* = 0.005; Fig 1E). In the glomerular compartment, ACE2 mRNA expression was lower in CKD compared to HLD (*P* = 0.0002; Fig 1B), due primarily to a difference between males and females in the CKD cohort (*P* = 0.04; Fig 1D) that was not observed in HLD (Fig 1F).

### Correlation of ACE2 mRNA expression with eGFR, age, and BMI in CKD

**Fig 2:**
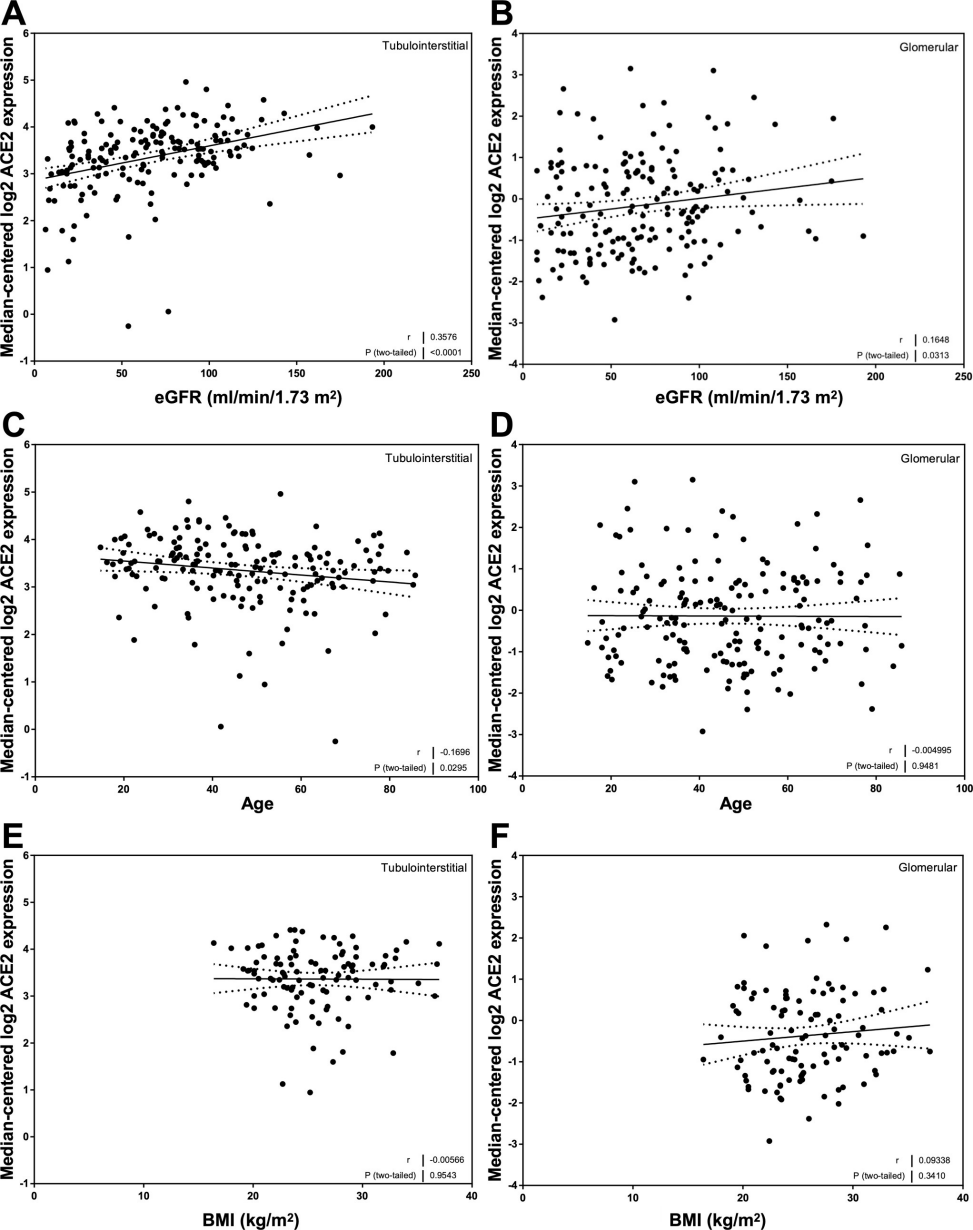
Correlation of tubular and glomerular Angiotensin-Converting Enzyme 2 (ACE2) mRNA expression with clinical variables in subjects with Chronic Kidney Disease (CKD). (A, C, E) Tubular ACE2 mRNA expression correlations with (A) estimated glomerular filtration rate (eGFR_MDRD_), (C) age, and (E) body mass index (BMI) in subjects with CKD. (B, D, F) Glomerular ACE2 mRNA expression correlations with (B) eGFR_MDRD_, (D) age, and (F) BMI in subjects with CKD. Pearson’s correlation coefficient (*r*) with two-tailed *P* values were calculated. Linear regression was used to generate the line of best fit (solid lines) with 95% confidence intervals (dotted lines).

Mean values for ACE2 mRNA expression in both the tubulointerstitial and glomerular compartments were similar in all of the disease categories represented in the CKD cohort (S1 Fig). There was a relationship between ACE2 mRNA expression and eGFR in the tubulointerstitium (*r* = 0.36, *P* < 0.0001; Fig 2A) and the glomerulus (*r* = 0.16, *P* = 0.03; Fig 2B). ACE2 mRNA expression was related to age in the tubulointerstitium (*r* = -0.17, *P* = 0.03; Fig 2C) but not glomeruli (Fig 2D). ACE2 mRNA expression was not related to BMI in either compartment (Fig 2E and 2F).

### Correlation of ACE2 mRNA expression with genes implicated in inflammation in CKD

**Fig 3:**
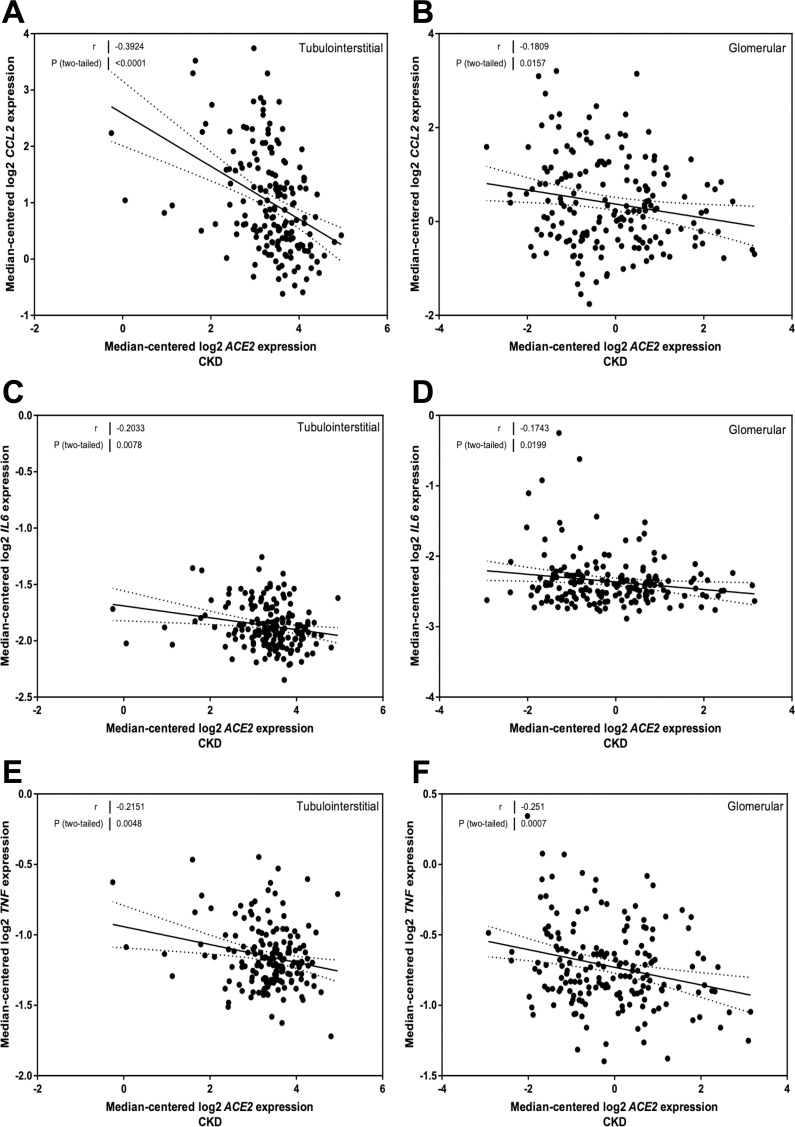
Correlation of tubular and glomerular Angiotensin-Converting Enzyme 2 (ACE2) mRNA expression with mRNA expression of inflammation genes in subjects with Chronic Kidney Disease (CKD). (A, B) Correlation of monocyte chemoattractant protein 1 (MCP-1 or *CCL2*) expression with ACE2 mRNA expression in the (A) tubulointerstitium and (B) glomeruli. (C, D) Correlation of interleukin 6 (*IL6*) expression with ACE2 mRNA expression in the (C) tubulointerstitium and (D) glomeruli. (E, F) Correlation of tumor necrosis factor alpha (*TNF*) expression with ACE2 mRNA expression in the (E) tubulointerstitium and (F) glomeruli. Pearson’s correlation coefficient (*r*) with two-tailed *P* values were calculated. Linear regression was used to generate the line of best fit (solid lines) with 95% confidence intervals (dotted lines).

CCL2 mRNA expression was related to ACE2 mRNA levels in the tubulointerstitium (*r* = -0.39, *P* < 0.0001; Fig 3A) and glomeruli (*r* = -0.18, *P* = 0.02; Fig 3B). IL6 mRNA expression was also related to ACE2 mRNA levels in the tubulointerstitium (*r* = -0.20, *P* = 0.008; Fig 3C) and glomeruli (*r* = -0.17, *P* = 0.02; Fig 3D). TNF mRNA levels were also related to ACE2 in the tubulointerstitial (*r* = -0.22, *P* = 0.005; Fig 3E) and glomerular (*r* = -0.25, *P* = 0.0007; Fig 3F) compartments.

### Correlation of ACE2 mRNA expression with genes implicated in fibrosis in CKD

**Fig 4:**
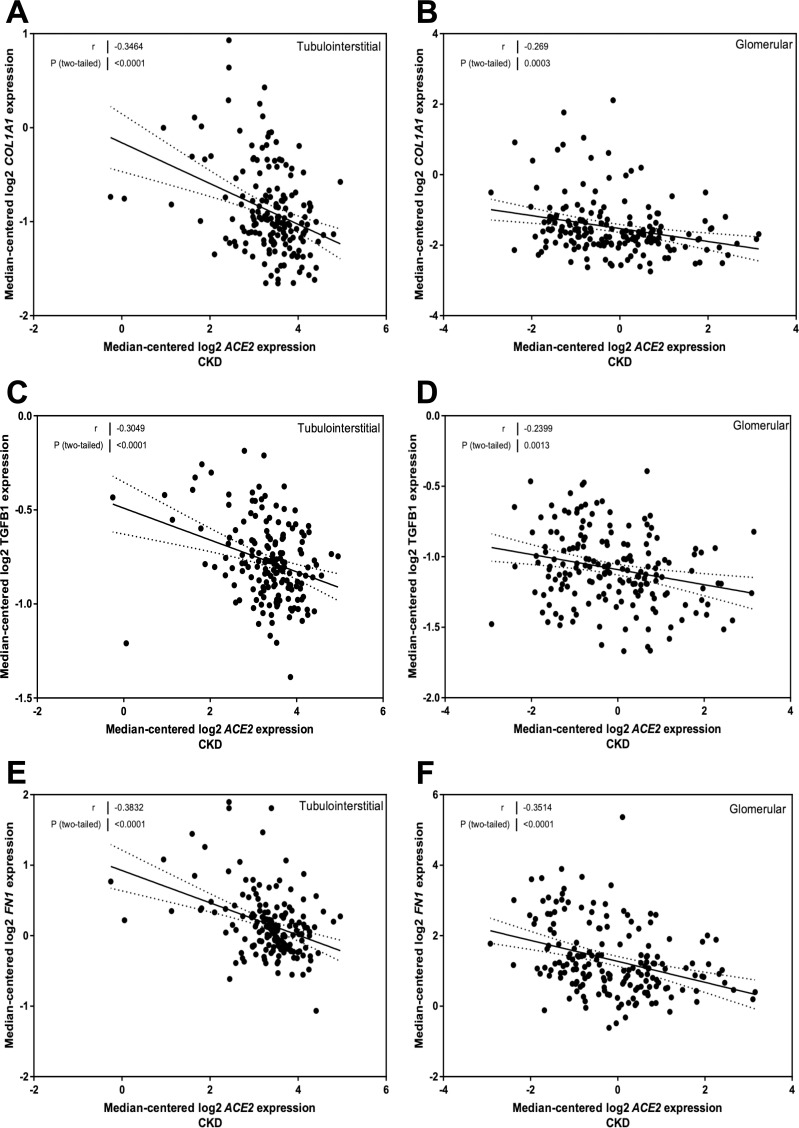
Correlation of tubular and glomerular Angiotensin-Converting Enzyme 2 (ACE2) mRNA expression with mRNA expression of fibrosis genes in subjects with Chronic Kidney Disease (CKD). (A, B) Correlation of collagen, type I, alpha 1 (*COL1A1*) expression with ACE2 mRNA expression in the (A) tubulointerstitium and (B) glomeruli. (C, D) Correlation of transforming growth factor beta 1 (*TGFB1*) expression with ACE2 mRNA expression in the (C) tubulointerstitium and (D) glomeruli. (E, F) Correlation of fibronectin (*FN1*) expression with ACE2 mRNA expression in the (E) tubulointerstitium and (F) glomeruli. Pearson’s correlation coefficient (*r*) with two-tailed *P* values were calculated. Linear regression was used to generate the line of best fit (solid lines) with 95% confidence intervals (dotted lines).

There was a relationship between COL1A1 mRNA expression and ACE2 mRNA levels in the tubulointerstitium (*r* = -0.35, *P* < 0.0001; Fig 4A) and glomeruli (*r* = -0.27, *P* = 0.0003; Fig 4B). TGFB1 mRNA expression was also related to ACE2 in the tubulointerstitium (*r* = -0.30, *P* < 0.0001; Fig 4C) and glomeruli (*r* = -0.24, *P* = 0.001; Fig 4D). FN1 mRNA expression was related to ACE2 in both the tubulointerstitial (*r* = -0.38, *P* < 0.0001; Fig 4E) and glomerular (*r* = -0.35, *P* < 0.0001; Fig 4F) compartments.

### Comparison of TMPRSS2 mRNA expression in HLD and CKD

**Fig 5:**
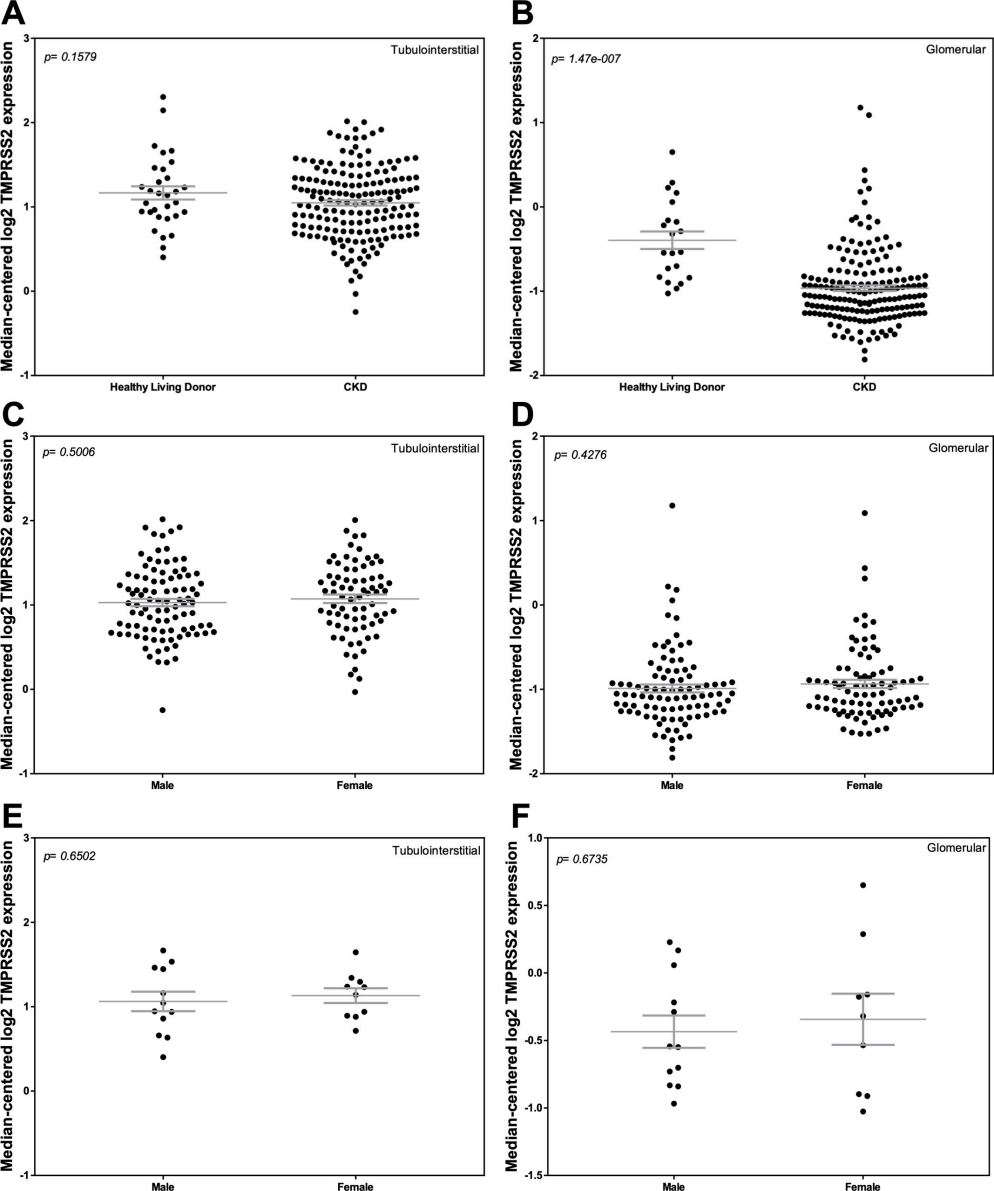
Tubular and glomerular transmembrane protease, serine 2 (TMPRSS2) mRNA expression in Chronic Kidney Disease (CKD) and Healthy Living Donors (HLD). (A, C, E) Tubular TMPRSS2 mRNA expression in (A) HLD and subjects with CKD, (C) males and females with CKD, and (E) male and female HLD. (B, D, F) Glomerular TMPRSS2 expression in (B) HLD and subjects with CKD, (D) males and females with CKD, and (F) male and female HLD. Values are the mean ± SEM (grey lines). *P* values were determined by Student’s *t* tests.

We compared TMPRSS2 mRNA expression between HLD and patients with CKD in both the glomerular and tubulointerstitial compartments. We also examined TMPRSS2 mRNA expression on the basis of sex. Tubulointerstitial TMPRSS2 mRNA expression was similar between the HLD and CKD groups (Fig 5A). In both HLD and patients with CKD, TMPRSS2 mRNA expression in the tubulointerstitium was similar in females and males (Fig 5C and 5E). In the glomerular compartment, TMPRSS2 mRNA expression was lower in CKD compared to HLD (*P* < 0.0001; Fig 5B), although there were no sex differences in either group (Fig 5D and 5F).

### Correlation of TMPRSS2 mRNA expression with eGFR, age, BMI, and ACE2 mRNA expression

**Fig 6:**
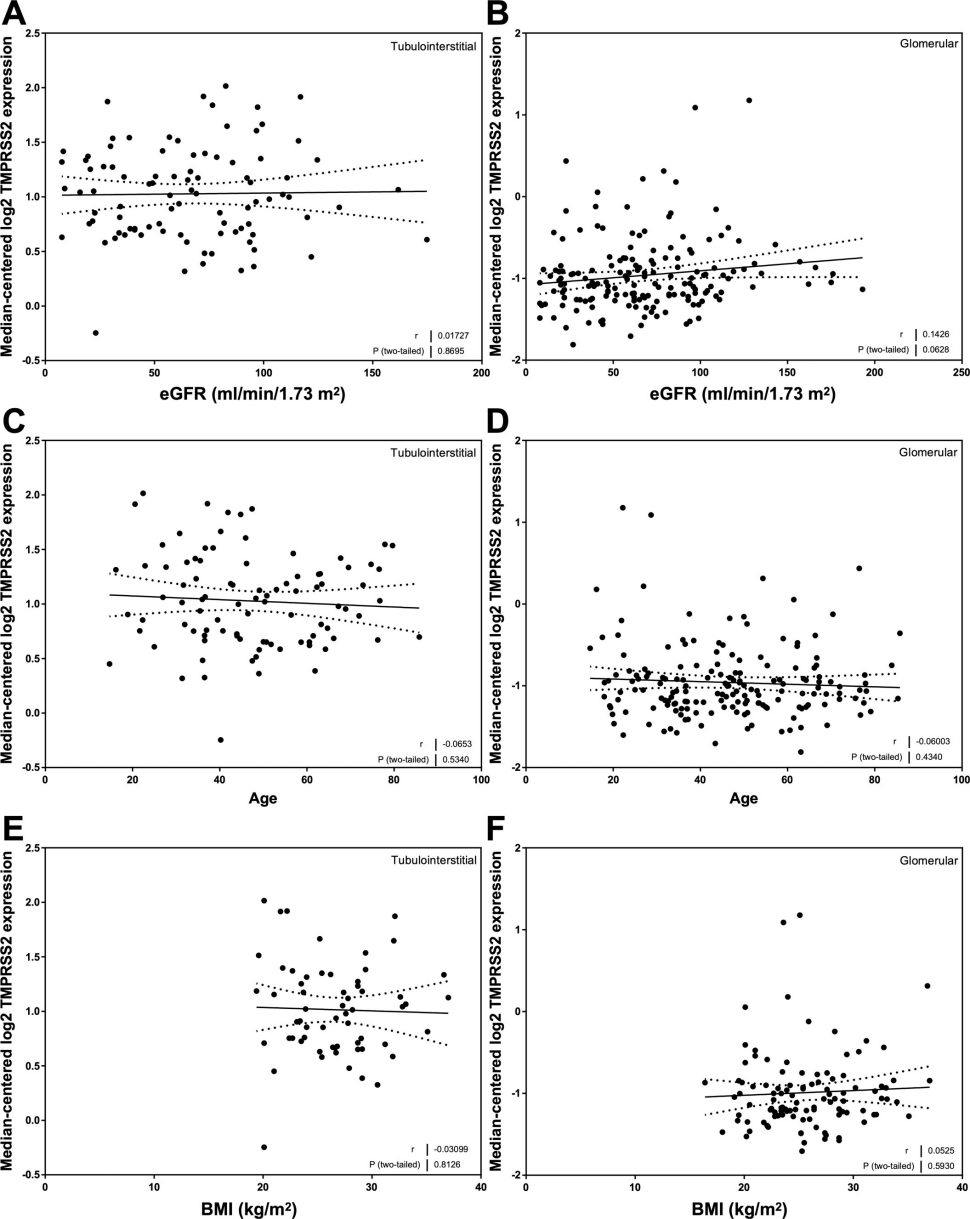
Correlation of tubular and glomerular transmembrane protease, serine 2 (TMPRSS2) mRNA expression with clinical variables in subjects with Chronic Kidney Disease (CKD). (A, C, E) Tubular TMPRSS2 mRNA expression correlations with (A) estimated glomerular filtration rate (eGFR_MDRD_), (C) age, and (E) body mass index (BMI) in subjects with CKD. (B, D, F). Glomerular TMPRSS2 mRNA expression correlations with (B) eGFR_MDRD_, (D) age, and (F) BMI in subjects with CKD. Pearson’s correlation coefficient (*r*) with two-tailed *P* values were calculated. Linear regression was used to generate the line of best fit (solid lines) with 95% confidence intervals (dotted lines).

**Fig 7:**
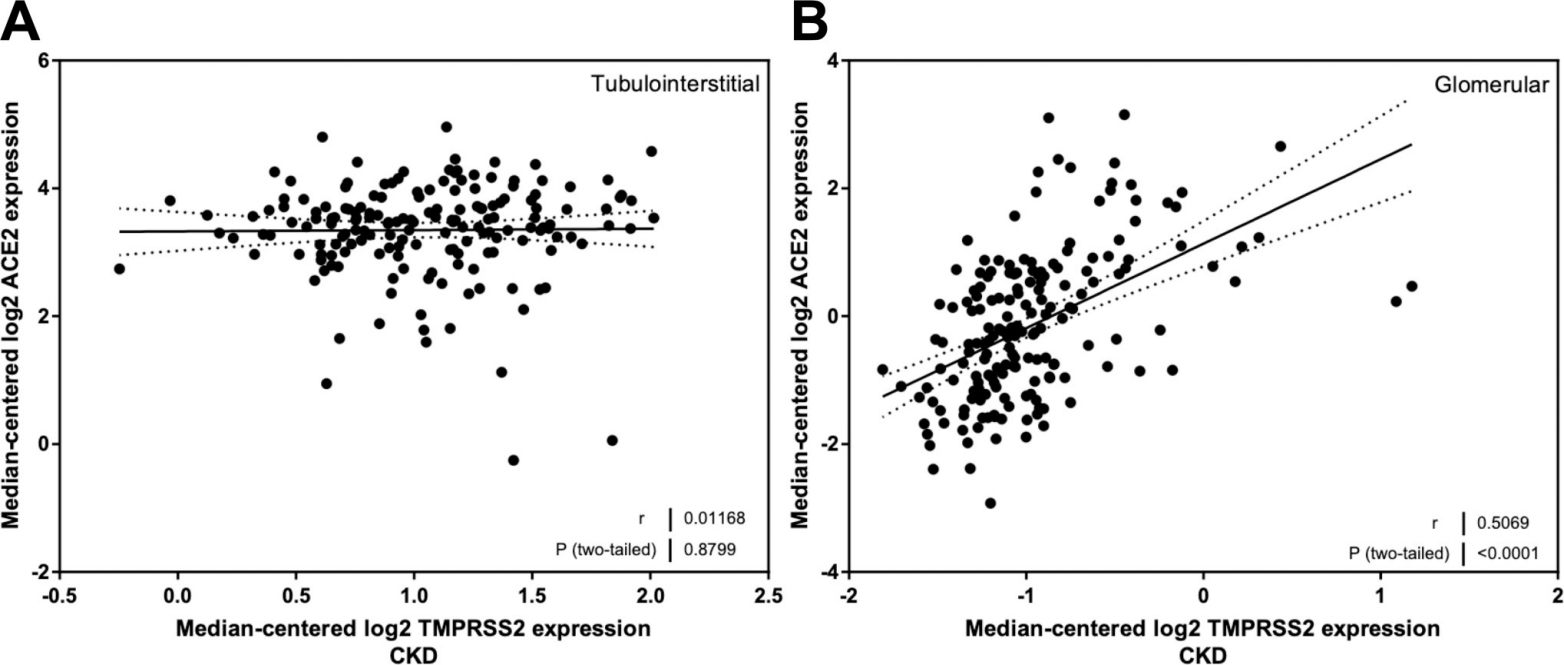
Correlation of tubular and glomerular transmembrane protease, serine 2 (TMPRSS2) mRNA expression with ACE2 mRNA levels in subjects with Chronic Kidney Disease (CKD). (A) Tubular TMPRSS2 mRNA expression correlation with ACE2 mRNA expression. (B) Glomerular TMPRSS2 mRNA expression correlation with ACE2 mRNA expression. Pearson’s correlation coefficient (*r*) with two-tailed *P* values were calculated. Linear regression was used to generate the line of best fit (solid lines) with 95% confidence intervals (dotted lines).

There were no relationships between TMPRSS2 mRNA expression and eGFR in the tubulointerstitium (Fig 6A) or glomerulus (Fig 6B). In a similar manner, TMPRSS2 mRNA expression was not related to either age (Fig 6C and 6D) or BMI (Fig 6E and 6F). There was no correlation between TMPRSS2 and ACE2 expression in the tubulointerstitial compartment (Fig 7A), but there was a strong positive correlation between ACE2 mRNA expression and TMPRSS2 mRNA expression in the glomerulus (*r* = 0.51, *P* < 0.0001; Fig 7B).

### Patient characteristics in the DN group

We compared the expression of ACE2 in the HLD to the subgroup of CKD subjects with DN. The DN tubulointerstitial cohort included 17 subjects with 5 females and 12 males (Table 3). The average age was 58.3 ± 2.6 years with a mean eGFR_MDRD_ of 44.3 ± 6.0 ml/min/1.73 m^2^. The DN glomerular cohort included 12 subjects (Table 3). There were 4 females and 8 males, and the average age was 54.8 ± 3.2 years with a mean eGFR_MDRD_ of 52.9.6 ± 8.8 ml/min/1.73 m^2^. Diabetic subjects had lower eGFR values compared to the HLD (*P* < 0.0001).

**Table 3:** Demographic and clinical characteristics of healthy living donors and subjects with diabetic nephropathy.

	Tubulointerstitium			Glomerulus		
	HLD	DN	*P* value (DN vs. HLD)	HLD	DN	*P* value (DN vs. HLD)
***n***	31	17		21	12	
**Sex (females/males)**	10/12	5/12	0.4906	9/12	4/8	0.8663
**Age (years)**	47.3 ± 2.4	58.3 ± 2.6	0.0042	47.2 ± 2.6	54.8 ± 3.2	0.0776
**BMI (kg/m**^**2**^**)**	23.7 ± 0.1	27.7 ± 1.3	0.2544	23.7 ± 0.1	27.0 ± 1.8	0.3675
**Mean BP (mm Hg)**	93.3 ± 10.7	105.8 ± 3.7	0.2349	93.3 ± 10.7	106.9 ± 4.1	0.2220
**SCR (mg/dl)**	0.82 ± 0.06	2.24 ± 0.39	0.0002	0.83 ± 0.06	1.83 ± 0.32	0.0004
**BUN (mg/dl)**	–	68.6 ± 17.9	–	–	58.9 ± 11.6	–
**eGFR (ml/min/1.73 m**^**2**^**)**	104.3 ± 6.5	44.3 ± 6.0	< 0.0001	105.4 ± 6.8	52.9 ± 8.8	< 0.0001

BMI, body mass index; BP, blood pressure; BUN, blood urea nitrogen; DN, diabetic nephropathy; eGFR, estimated glomerular filtration rate; HLD, healthy living donor; SCR, serum creatinine. For continuous variables, values are presented as the mean ± SEM and *P* values were determined by Student’s *t* tests. For categorical variables, *P* values were determined by χ^2^ tests.

### Comparison of ACE2 mRNA expression in HLD and DN

**Fig 8:**
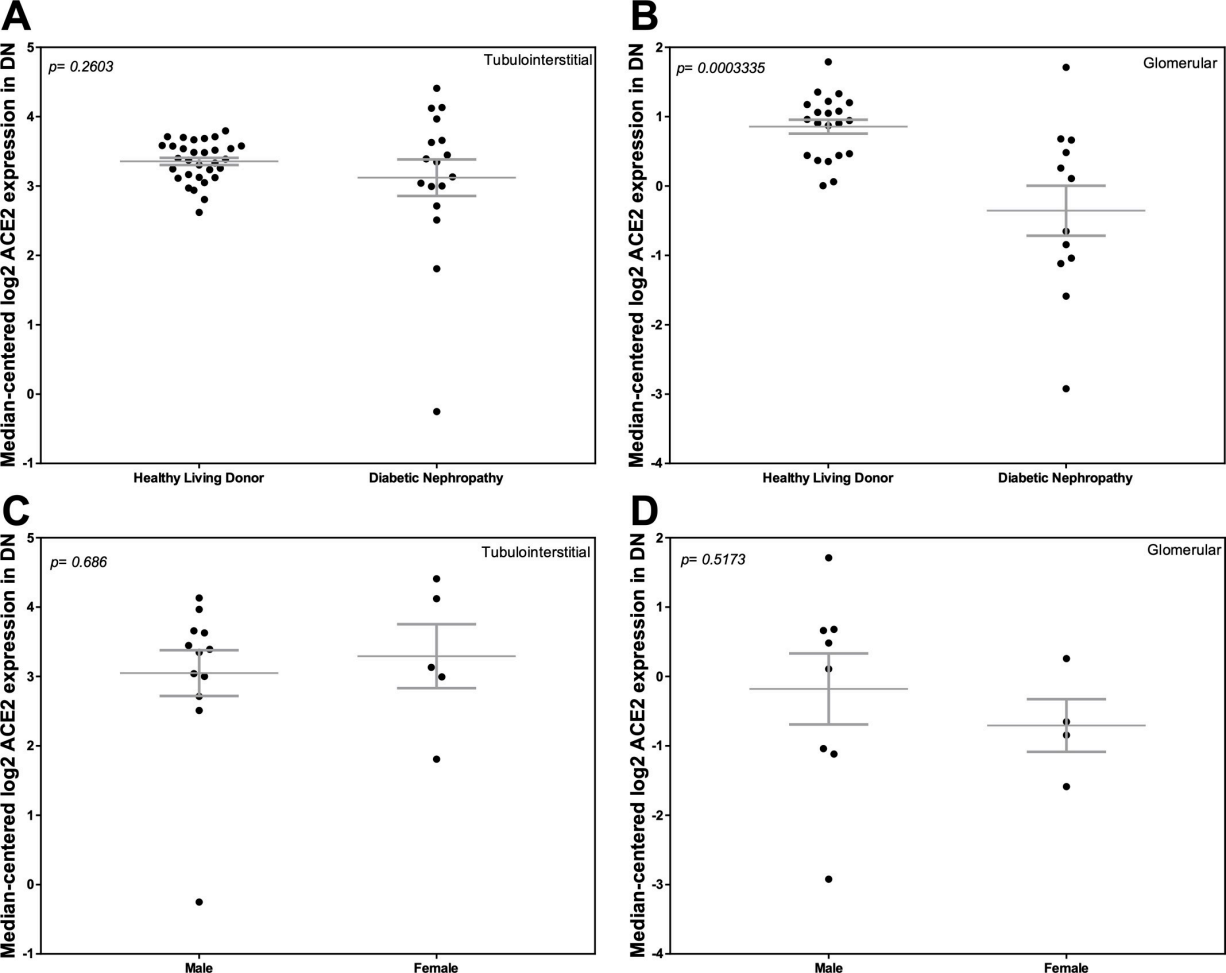
Tubular and glomerular Angiotensin-Converting Enzyme 2 (ACE2) mRNA expression in Diabetic Nephropathy (DN) and Healthy Living Donors (HLD). (A, C) Tubular ACE2 mRNA expression in (A) HLD and subjects with DN, and (C) male and female subjects with DN. (B, D) Glomerular ACE2 mRNA expression in (B) HLD and subjects with DN, and (D) male and female subjects with DN. Values are the mean ± SEM (grey lines). *P* values were determined by Student’s *t* tests.

We compared ACE2 mRNA expression in both kidney compartments in subjects with DN and HLD. In subjects with DN, we also explored whether ACE2 mRNA expression differed between females and males. In the tubulointerstitium, ACE2 mRNA expression was similar between the two groups (Fig 8A). Glomerular ACE2 mRNA expression was lower in DN compared to HLD (*P* = 0.0003; Fig 8B). ACE2 expression in either kidney compartment was similar between males and females with DN (Fig 8C and 8D).

### Correlation of ACE2 mRNA expression with eGFR, age, and BMI in DN

**Fig 9:**
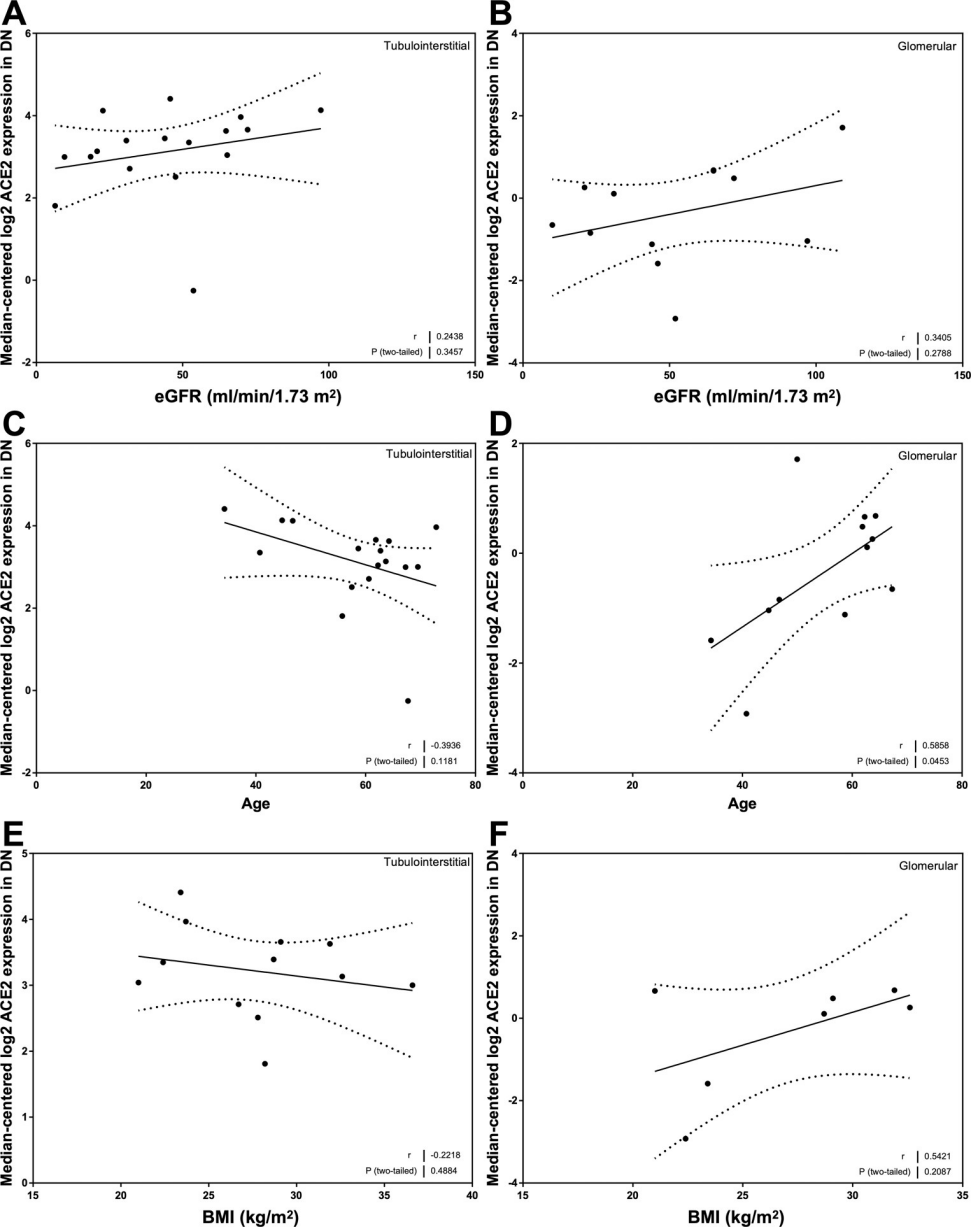
Correlation of tubular and glomerular Angiotensin-Converting Enzyme 2 (ACE2) mRNA expression with clinical variables in subjects with Diabetic Nephropathy (DN). (A, C, E) Tubular ACE2 mRNA expression correlations with (A) estimated glomerular filtration rate (eGFR_MDRD_), (C) age, and (E) body mass index (BMI) in subjects with DN. (B, D, F) Glomerular ACE2 mRNA expression correlations with (B) eGFR_MDRD_, (D) age, and (F) BMI in subjects with DN. Pearson’s correlation coefficient (*r*) with two-tailed *P* values were calculated. Linear regression was used to generate the line of best fit (solid lines) with 95% confidence intervals (dotted lines).

In both the tubulointerstitial (Fig 9A) and glomerular (Fig 9B) compartments, there were weak positive associations between ACE2 expression and eGFR, although neither reached statistical significance, likely based, in part, on the number of subjects available for analysis (*n* = 17 for the tubulointerstitium and *n* = 12 for the glomerulus). Opposing trends were observed in the tubulointerstitial and glomerular compartments regarding the relationships between ACE2 expression and age (Fig 9C and 9D) and ACE2 expression and BMI (Fig 9E and 9F), but only the relationship between ACE2 expression and age in the glomerulus reached statistical significance (*r* = 0.59, *P* < 0.05; Fig 9D).

### Correlation of ACE2 mRNA expression with genes implicated in inflammation and fibrosis in subjects with DN

**Fig 10:**
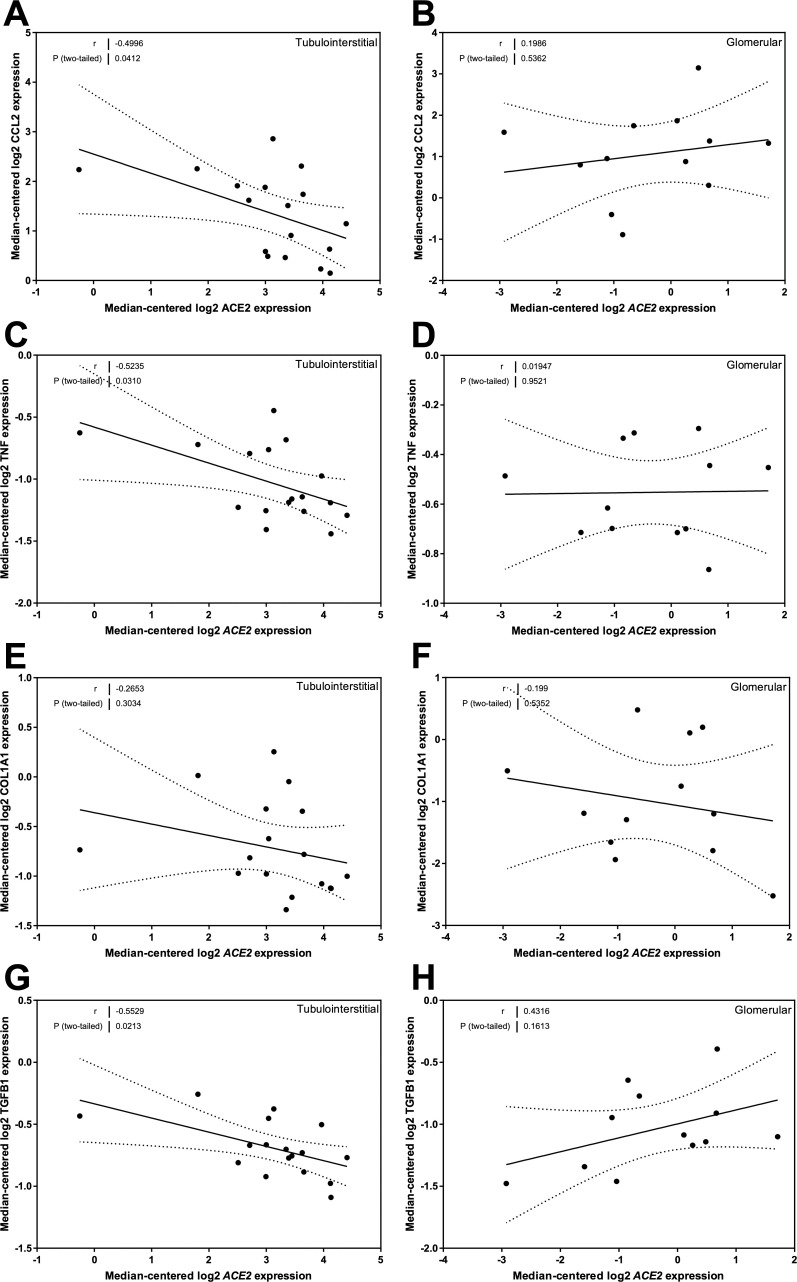
Correlation of tubular and glomerular Angiotensin-Converting Enzyme 2 (ACE2) mRNA expression with mRNA expression of inflammation and fibrosis genes in subjects with Diabetic Nephropathy (DN). (A, B) Correlation of monocyte chemoattractant protein 1 (MCP-1 or *CCL2*) expression with ACE2 mRNA expression in the (A) tubulointerstitium and (B) glomeruli. (C, D) Correlation of tumor necrosis factor alpha (*TNF*) expression with ACE2 mRNA expression in the (C) tubulointerstitium and (D) glomeruli. (E, F) Correlation of collagen, type I, alpha 1 (*COL1A1*) expression with ACE2 mRNA expression in the (E) tubulointerstitium and (F) glomeruli. (G, H) Correlation of transforming growth factor beta 1 (*TGFB1*) expression with ACE2 mRNA expression in the (G) tubulointerstitium and (H) glomeruli. Pearson’s correlation coefficient (*r*) with two-tailed *P* values were calculated. Linear regression was used to generate the line of best fit (solid lines) with 95% confidence intervals (dotted lines).

CCL2 mRNA expression was inversely correlated to ACE2 mRNA levels in the tubulointerstitium (*r* = -0.50, *P* = 0.04; Fig 10A) but not in glomeruli (Fig 10B) of patients with DN. TNF mRNA expression was also related to ACE2 mRNA levels in the tubulointerstitium (*r* = -0.52, *P* = 0.03; Fig 10C) but not in glomeruli (Fig 10D). There were negative correlations between ACE2 expression and COL1A1 expression in both the tubulointerstitial (Fig 10E) and glomerular (Fig 10F) compartments, although neither reached statistical significance. Interestingly, opposing trends were seen in the relationships between ACE2 expression and TGFB1 mRNA expression in the tubulointerstitial (Fig 10G) and glomerular (Fig 10H) compartments. The relationship between ACE2 and TGFB1 mRNA expression in the tubulointerstitium reached statistical significance (*r* = -0.55, *P* = 0.02; Fig 10G).

## Discussion

ACE2, found mainly in the brush border of the proximal tubule and the glomerular podocyte, is a determinant of experimental kidney injury [1–3, 33]. There are few studies of ACE2 expression in CKD in humans [26, 28, 29], and interestingly, studies have shown that ACE2 is a receptor for SARS-CoV and SARS-CoV-2 [12, 34]. Accordingly, we accessed publicly available data from the ERCB (nephroseq.org) to study the effect of CKD on ACE2 expression and to examine the relationships between clinical variables and ACE2 expression. Given the role of the membrane-bound protease, TMPRSS2, along with ACE2, in SARS-CoV-2 infection [12, 13], we also investigated TMPRSS2 expression in the glomerular and tubulointerstitial compartments of the kidney. In addition, loss of ACE2 accelerates the progression of experimental kidney disease [35–37]; therefore, we also studied the relationships between kidney ACE2 expression and genes implicated in inflammation and fibrosis in CKD.

Our first major observation was that mean levels of ACE2 expression were similar in the kidney tubulointerstitium in CKD and HLD but lower in glomeruli in CKD. There was considerable variability in CKD, likely due, in part, to the variety of kidney pathologies in the CKD cohort. We then examined the impact of sex on ACE2 expression. ACE2 expression was lower in males than in females in both kidney compartments. The *ACE2* gene is located on the X chromosome [38], but X inactivation should limit any effect on ACE2 expression. Male sex is a risk factor for the progression of CKD [39]. Although generally attributed to the effect of sex hormones on kidney cells, differences in ACE2 expression may also contribute, at least in part, to this differential risk.

Our second set of observations was related to the associations between ACE2 expression and clinical variables. We first examined the relationship between ACE2 expression and eGFR in the CKD cohort. Lower values of eGFR were associated with lower levels of ACE2 expression in the kidney. This relationship was more marked in the tubulointerstitium than in glomeruli. We next examined the relationship between age and ACE2 expression in the kidney and focused on the CKD cohort for this analysis. There was a very modest negative correlation between age and ACE2 expression in the tubulointerstitium and no relationship in glomeruli. This finding contrasts with reports that showed an age-dependent increase in upper and lower respiratory tract ACE2 gene expression [40, 41]. Finally, we did not see any relationship between BMI and ACE2 expression in the kidney in the CKD cohort; although we cannot rule out a threshold effect for BMI [42, 43], especially in a healthy population. Taken together, these findings suggest that the impact of eGFR and age on ACE2 expression is more important in the tubulointerstitial compartment than in the glomerular compartment.

We found that tubulointerstitial TMPRSS2 mRNA expression was similar in the kidneys of HLD and subjects with CKD, but it was lower in the glomerular compartment of CKD subjects than in HLD. There was no impact of sex, eGFR, age, or BMI on expression in either compartment. There was a strong relationship between ACE2 mRNA expression and TMPRSS2 mRNA expression in glomeruli. Batlle and coworkers reported single-cell RNA expression of ACE2 and TMPRSS2 in the kidney [33]. They found that ACE2 was predominantly expressed in proximal tubule cells. Li *et al*. have reported that ACE2 is also expressed in the glomerulus [44]. In contrast, Batlle and coworkers found that TMPRSS2 expression was greatest in the distal convoluted tubule and type A intercalated cells of the collecting duct, with modest expression in other cell types, including the podocyte. This cell-specific pattern of expression may account for our observation that there was no correlation between ACE2 and TMPRSS2 expression in the tubulointerstitium.

It has been postulated that co-expression of ACE2 and TMPRSS2 underlies the susceptibility of other tissues to infection [14]. The colocalization and co-expression of ACE2 and TMPRSS2 in the glomerulus and their strong correlation in this compartment may underlie the recent clinical observation of an emerging SARS-CoV-2–associated nephropathy (COVAN) which is characterized by a collapsing form of glomerulosclerosis not unlike HIV-associated nephropathy [45]. Notwithstanding these observations, it is important to note that we did not study ACE2 expression in subjects that were infected with SARS-CoV-2.

We next examined the relationships between ACE2 expression and genes implicated in inflammation and fibrosis. The rationale was that ACE2 reduces inflammation and fibrosis by limiting Ang II and promoting Ang-(1–7). In kidneys of mice with ischemia-reperfusion injury or unilateral ureteral obstruction, the loss of ACE2 exacerbated infiltration of neutrophils, F4/80^+^ macrophages, and CD3^+^ T cells, and increased mRNA levels of cytokines and chemokines, including IL6, TNF, and MCP-1 [35, 36]. Moreover, Ang-(1–7) has been shown to reduce expression of IL6 and TNF in macrophages [46]. Our third major observation was that there were significant relationships between ACE2 mRNA expression and mRNA expression of CCL2, IL6, and TNF in both the tubulointerstitium and glomeruli of the CKD cohort. Studies have also shown that ACE2 limits kidney fibrosis and ACE2 deficiency was associated with higher expression of COL1A1, TGFB1, and FN1 in mice [36, 37]. Lower levels of ACE2 mRNA expression were related to increased mRNA expression of COL1A1, TGFB1, and FN1. Taken together with experimental studies of ACE2 null mice [37, 47–49] and studies of recombinant ACE2 treatment [50–52], these findings suggest that low ACE2 expression may contribute to the progression of CKD, by enhancing early inflammation and contributing to long-term fibrosis.

Finally, we performed a subgroup analysis of the subjects with DN. We saw a trend towards decreased ACE2 expression in the tubulointerstitium and a statistically significant decrease in glomerular ACE2 mRNA in DN compared to HLD. These findings confirm our original observations in DN [26]. In our previous study, we used laser-capture microdissection to examine ACE2 expression in both the glomerular and tubulointerstitial compartments. Diabetes was associated with decreased ACE2 expression in both kidney compartments [26]. More recently, Gilbert and coworkers reported increases in kidney ACE2 mRNA expression in patients with DN [53], a finding that may reflect the use of formalin-fixed paraffin-embedded biopsy cores that were not microdissected.

We then extended our studies of the DN subset to look at the relationships between ACE2 mRNA expression and eGFR, age, and BMI. There was a positive association between glomerular ACE2 expression and age in DN. Interestingly, age appears to be a determinant of renin-angiotensin system (RAS) activation in the kidney [54]. The relationships between ACE2 expression and the other clinical variables were not statistically significant, likely due, in part, to the fewer number of subjects that we were able to analyze. However, decreased ACE2 expression in the tubulointerstitium was associated with increased CCL2, TNF, and TGFB1 mRNA expression. Tissue depletion of ACE2 may promote inflammation and fibrosis in DN.

Our study has several limitations. First, although we were able to associate higher kidney ACE2 expression with lower expression of inflammation and fibrosis genes, it is important to note that our findings do not imply causation. Second, a wide range of underlying kidney pathologies was included in the CKD cohort, limiting our ability to look at disease-specific relationships. There were relatively few specimens (31 tubulointerstitial and 21 glomerular samples) in the HLD cohort, and this limited the analysis of relationships between ACE2 expression and age and BMI. Important clinical data regarding ethnicity and race, and treatment with ACEIs and/or ARBs were not available, so we could not address the impact of these variables on kidney ACE2 expression in our study. Finally, our study focused exclusively on ACE2 and TMPRSS2 mRNA expression and did not address protein expression or enzyme activity.

In conclusion, kidney ACE2 and TMPRSS2 mRNA expression differs in HLD and CKD. ACE2 expression is greater in females than in males in the tubulointerstitium and glomeruli, whereas there is no impact of sex on TMPRSS2 expression. There is a relationship between ACE2 expression and eGFR in CKD. ACE2 and TMPRSS2 expression levels are strongly correlated in the glomerulus but not in the tubulointerstitium. Finally, lower ACE2 expression is associated with higher expression of genes implicated in inflammation and fibrosis in both kidney compartments, suggesting that loss of ACE2 may promote inflammation and fibrosis in the tubulointerstitium and glomerulus.

## Supporting information

S1 Fig Tubular and glomerular Angiotensin-Converting Enzyme 2 (ACE2) mRNA expression in Chronic Kidney Disease (CKD) cohorts and Healthy Living Donors (HLD). (A) Tubular ACE2 mRNA expression in CKD cohorts and HLD. (B) Glomerular ACE2 mRNA expression in CKD cohorts and HLD. Values are the mean ± SEM. *P* values were determined by one-way analysis of variance.
(TIF)

S1 Dataset Clinical characteristics and pre-processed log2 median-centered microdissected tubulointerstitial and glomerular biopsy microarray data from the European Renal cDNA Bank. This file includes information from the Ju CKD TubInt and Ju CKD Glom datasets available from Nephroseq (nephroseq.org).
(XLSX)
