# Supplementary figures and images for: Kidney ACE2 expression: Implications for chronic kidney disease
Source: PLoS One. 2020 Oct 30;15(10):e0241534. doi: 10.1371/journal.pone.0241534 (PMC7598523; doi:10.1371/journal.pone.0241534)

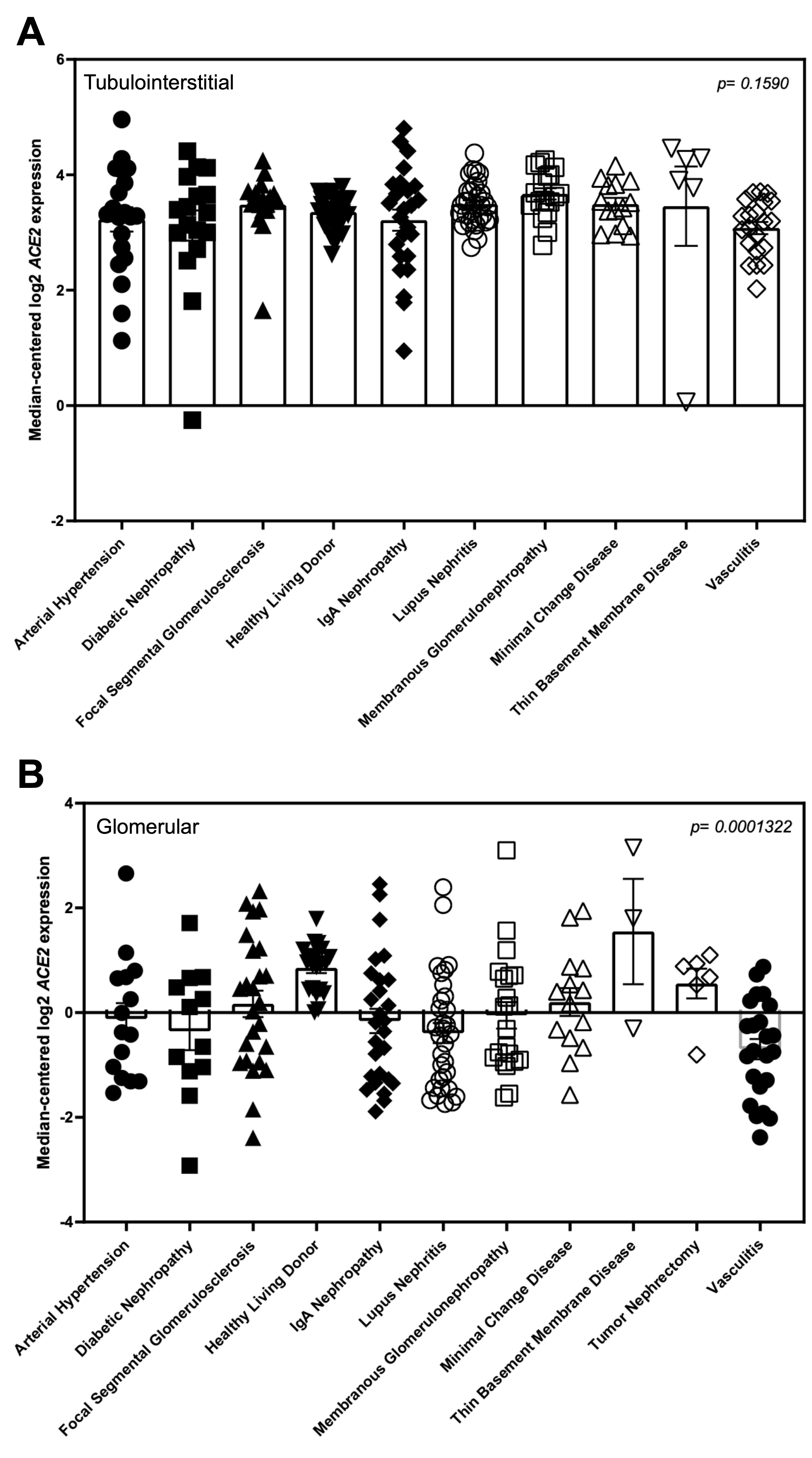

Supplement: S1 Fig — (A) Tubular ACE2 mRNA expression in CKD cohorts and HLD. (B) Glomerular ACE2 mRNA expression in CKD cohorts and HLD. Values are the mean ± SEM. P values were determined by one-way analysis of variance. (TIF) [file pone.0241534.s001.tif]
